# Effects of Substance Use, Recovery, and Non–Drug-Related Online Community Participation on the Risk of a Use Episode During Remission From Opioid Use Disorder: Longitudinal Observational Study

**DOI:** 10.2196/36555

**Published:** 2022-08-22

**Authors:** Elahe Naserianhanzaei, Miriam Koschate-Reis

**Affiliations:** 1 Department of Political Sciences, Faculty of Humanities, Arts and Social Sciences University of Exeter Exeter United Kingdom; 2 Department of Psychology, Faculty of Health and Life Sciences University of Exeter Exeter United Kingdom; 3 Institute for Data Science and Artificial Intelligence University of Exeter Exeter United Kingdom

**Keywords:** online communities, opioid addiction, recovery capital, social identity, Reddit, social media

## Abstract

**Background:**

Opioid addiction is currently one of the most pressing public health issues. Despite several treatment options for opioid addiction, the recurrence of use episodes during remission remains high. Research indicates that meaningful membership in various social groups underpins the successful transition from addiction to long-term remission. However, much of the current literature focuses on online peer-support groups for individuals in remission from substance use, sometimes also called recovery groups, a term we will use in line with the terminology used by the online community we studied. In contrast, online group memberships that promote substance use and groups that are unrelated to substance use and remission (non–drug-related groups) are rarely studied.

**Objective:**

This study aims to understand whether engagement with a variety of Reddit subforums (subreddits) provides those in remission from opioid use disorder (OUD) with social capital, thereby reducing their risk of a use episode over several years. More specifically, it aims to examine the different effects of engagement with substance use, recovery, and non–drug-related subreddits.

**Methods:**

A data set of 457 individuals in remission from OUD who posted their remission start date on Reddit was collected, of whom 219 (47.9%) indicated at least one use episode during the remission period. Using a Cox proportional hazards model, the effects of the number of non–drug-related, recovery, and substance use subreddits an individual had engaged with on the risk of a use episode were tested. Group engagement was assessed both in terms of the absolute number of subreddits and as a proportion of the total number of subreddits in which an individual had posted.

**Results:**

Engagement with a larger number of non–drug-related online communities reduced the likelihood of a use episode irrespective of the number of posts and comments made in these forums. This was true for both the absolute number of non–drug-related communities (*P*<.001) and the proportion of communities with which a person engaged (*P*<.001). The findings were less conclusive for recovery support and substance use groups; although participating in more recovery support subreddits reduced the risk of a use episode (*P*<.001), being part of a higher proportion of recovery support groups relative to other subreddits increased the risk (*P*=.01). A higher proportion of substance use subreddits marginally increased the risk of a use episode (*P*=.06); however, the absolute number of substance use subreddits significantly reduced the risk of a use episode (*P*=.002).

**Conclusions:**

Our work indicates that even minimal regular engagement with several non–drug-related online forums may provide those in remission from OUD with an opportunity to grow their social capital and reduce the risk of a use episode over several years.

## Introduction

### Background

In recent years, research on remission from addiction has shown the importance of social groups [1,2] and recovery capital [3] more widely. This trend builds on recent findings in the health and well-being literature, which suggest that the joining of new groups can act as a *social cure* [4]. The social cure hypothesis states that a higher number of memberships in social groups is associated with better mental and physical health outcomes, better resilience, and higher well-being. With regard to substance use disorders, much of the literature focuses on the benefits of peer-support groups for remission. Overall, this literature suggests that highly structured, peer-led support groups, both offline and online, support remission [5-10]. Few studies have examined the impact of social groups beyond such peer-support groups, and these tend to focus on a small number of offline groups or individuals (eg, family, friends, and coworkers) [11,12], thereby overlooking the potential of *online communities* as social capital for managing the remission process.

Understanding the impact of online community memberships beyond the direct effects of online peer-support groups can help tailor support for those with limited access to offline social networks. Access to a face-to-face support network can be limited by geographical location (eg, rural areas), difficulties with mobility (eg, lack of transport and failure of groups to accommodate mobility impairments), or significant caring responsibilities (eg, childcare availability) [13,14]. In some cases, the stigma surrounding addiction can also make it harder for those in remission to access offline help [15]. More recently, lockdowns during the COVID-19 pandemic have severely limited face-to-face interactions for those in remission, not only with peer-support groups but also with a wider support network of family, friends, and health care professionals [16]. Therefore, it is not surprising that online communities play an increasingly important role in providing social support, advice, and information [17,18] and may act as an additional source of social capital during remission.

### Social Groups and Recovery

#### Overview

Research on the social cure provides mounting evidence that membership in several social groups increases health, well-being, and resilience [4,19]. For instance, research shows that a higher number of self-reported group memberships is associated with lower levels of smoking and drinking [20]. A recent study of online communities found that the number of subreddits an individual posted in and the evenness of participation in these different online communities reduced the risk of use episodes over several years for those in remission from opioid use disorder (OUD) [21]. Being part of various social groups is thought to provide members with psychological and physical resources such as a sense of connectedness, meaning, purpose, and worth as a member of a positively valued group [4]. Furthermore, groups also provide direct and indirect social support, a sense of personal control through the group’s ability to affect change, and the social power and agency that the opportunity for collective action brings [4,22]. Similarly, recovery capital can be built by developing and strengthening links with those who are in recovery (*bonding*) and the wider community that may provide support (*bridging*) [3,23,24].

The Social Identity Model of Recovery (SIMOR) [1] integrates these 2 strands of research and suggests that meaningful membership in various social groups underpins the successful transition from addiction to long-term recovery. Rather than focusing on a few strong interpersonal bonds, the SIMOR suggests that being a part of several social groups is among the key factors associated with remission from substance use. Group membership becomes part of an individual’s self-concept—their social identity—once the individual sees themselves, and is seen by others, as belonging to a particular social group or category [25,26]. When an individual identifies themselves as a group member, as well as begins to feel part of the group, group norms become internalized and guide attitudes, emotions, and behavior [27].

On the basis of this approach, being a member of a community that endorses substance use can be expected to increase substance use as this behavior is a normative expression of this particular group membership. In contrast, the model suggests that membership in recovery support groups shifts the identity away from an *addiction* identity toward a *recovery* identity. A *recovery* identity is thought to promote remission, such as a reduction in substance use or abstinence, through its norms and by providing (social) resources. In the longer term, gaining or regaining membership in groups that are not associated with substance use or remission (ie, non–drug-related groups) should help the individual build a social identity that increases health and well-being and is resilient to life changes [28,29]. Hence, the SIMOR conceptualizes remission from substance use disorder as a long-term transformation of social relationships and, correspondingly, the social self. The self is transformed from an *addiction* identity to a *recovery* identity toward a range of non–drug-related social identities (eg, parent, employee, and volunteer) that the individual gains or regains during their recovery journey [1].

#### Recovery Communities

Joining and participating in traditional support groups, such as Alcoholics Anonymous (AA) or Narcotics Anonymous, have shown significant promise in assisting individuals with a substance use disorder in maintaining their abstinence [30] by providing an encouraging and supportive community and by facilitating programs for addiction management and remission. An AA intervention designed to change an individual’s social network—away from network members encouraging substance use toward abstinence-supporting network members—found positive effects on behavioral and attitudinal support for abstinence [5,31].

Similarly, positive effects on sustained remission have been found for online peer-support networks. For example, research found that engagement in online recovery support communities on Reddit reduced the risk of recurring use episodes in those who were in remission from smoking or from alcohol use disorders [8]. Similarly, a qualitative study showed that participation in the online recovery support group *Soberistas* was related to an offline commitment to changing drinking behaviors [32]. Studies on recovery from OUD show that online recovery groups are supportive [33] and promote remission despite a high chance of a recurring use episode among group members [34].

Active participation has been shown to be an important component [8]. For instance, a recent study found that those who participated more on a Facebook page dedicated to supporting those with a substance use disorder in the early stages of remission were more likely to remain in the program [9]. Here, the level of participation was assessed through the number of received *likes* and increased use of the word *we*.

#### Non–Drug-Related Communities

Further evidence from the field of addiction highlights the important role of a wider social network beyond recovery support groups. Much of this literature examines the effect of peer support on the decision to "quit" substance use and start therapy [35,36] or the reverse effect of the decision to quit on the composition of the social network [37]. However, a few studies have shown that social network composition affects subsequent substance use.

A longitudinal social network study across 32 years found that alcohol consumption tends to follow the behavior of individuals in a person’s social network [38]. These effects were mostly driven by interpersonal relationships with family members and close friends rather than groups (eg, coworkers or neighbors). The study also did not differentiate between problematic alcohol consumption and general alcohol consumption.

Furthermore, a self-report study with residents in a therapeutic community found that a higher proportion of non–drug-related group memberships decreased substance use at the 6-month follow-up [11]. In this study, the number of group memberships and categorization into non–drug-related (or *low-risk*) and substance use (or *high-risk*) groups were based on a mapping exercise. Participants were asked to group their social relations into different categories (eg, family, friends, coworkers, and recovery peer groups). Participants also indicated the number of people in each *group* who regularly used substances. Groups in which most individuals used substances regularly were then labeled as *high-risk* groups and those in which most of the members were abstinent, in remission, or whose drug use was unknown to the participant were then labeled as *low-risk* groups, with more mixed groups remaining uncategorized. As a result, recovery support groups were not considered in their own right but were classified as high risk, low risk, or uncategorized based on the perceived substance use of its members and not based on the norms of the group. This method also does not clearly differentiate between interpersonal relations (eg, family and friends) and group membership (eg, work team). Surprisingly, participants indicated only a very small number of *group memberships* (median 4). This suggests that small groups were mostly considered by participants rather than wider social categories (eg, parent and Christian) or shared interest groups (eg, volunteer and rugby fan) that form an individual’s social identity [27].

In summary, current research suggests that face-to-face interactions with individuals and groups who are not engaging in substance use are more likely to support remission than interactions with individuals who are known to continue to use substances. However, there appears to be very little, if any, research examining the effects of a wide variety of (online) communities that are concerned with non–drug-related interests, such as video games, literature, sports, and politics. Instead, current literature focuses on small groups and interpersonal social networks.

#### Substance Use Communities

In line with the SIMOR, a few studies indicate that a higher proportion of individuals engaging in substance use—or substance use groups—in an individual’s network tends to be related to higher substance use [11,38], although others have found no statistically significant effect [39]. However, these studies also indicate difficulties in neatly categorizing a group as a *substance use group* as opposed to a *non–drug-related group*. The use versus nonuse binary has been challenged by several researchers (eg, [40,41]) as adding a burden on those in remission. For instance, qualitative research suggests that severing ties with groups that continue to engage in substance use can result in the loss of trusted and emotionally significant relationships, particularly for young people [40]. Importantly, those who continue to engage in substance use do not necessarily seek to undermine remission in others and may provide information and emotional support. Similarly, the suggestion that the "bad company" of substance use groups undermines remission has been criticized as being based on relatively sparse empirical evidence [41]. Specifically, for online communities, we are not aware of any research examining the effect of participation in communities that actively promote or endorse substance use on the risk of recurring use episodes for those in remission.

In summary, there appears to be some empirical support for the positive effects of recovery support groups and offline non–drug-related groups on remission from substance use disorders. However, evidence is surprisingly sparse regarding the effects of groups that promote substance use and non–drug-related online interest groups. Looking closely at the research also reveals a mix of approaches in determining the type of group—often revealing a focus on the perceived behavior of known individuals rather than the norms that the wider social group is promoting. It is also unclear from the current literature whether it is close interpersonal network members (eg, family, friends, and coworkers) or interactions with wider communities (eg, parents, volunteers, sports fans, and Christians) that support remission. Finally, the current literature is limited to separately examining the effects of substance use, recovery, and non–drug-related groups.

### Aim and Hypotheses

This study aimed to extend a previous study [21] on the effects of multiple online group memberships on remission from OUD. More specifically, this study tested the effects of substance use, recovery, and non–drug-related online communities separately in terms of absolute numbers of memberships and as a proportion of all memberships. Differentiating between the effects of different types of communities is both theoretically and practically important. In addition to testing the wider social cure hypothesis, this approach will test the SIMOR, which suggests that substance use groups affect remission differently from recovery and nonusing groups. Although several studies have tested parts of the SIMOR, only one study that we are aware of has tested all 3 types of groups together [11], and none has done so in an online environment. Understanding how different types of online communities affect remission also has practical implications by allowing those in remission to make more informed choices about their online community engagement. Furthermore, by examining data across several years, the study also sheds light on the longitudinal effects that these 3 different types of social groups have on the risk of a use episode over time, thereby considerably extending the knowledge about the long-term effects of social group membership.

In line with the SIMOR, we expected that a higher number (and proportion) of online community memberships actively supporting recovery will decrease the risk of a use episode over time (*hypothesis 1*). Similarly, a higher number (and proportion) of non–drug-related online groups were predicted to reduce the risk of a use episode (*hypothesis 2*). In contrast, a higher number (and proportion) of memberships in online communities that advocate substance use were expected to increase the risk of a use episode in those in remission from OUD (*hypothesis 3*).

## Methods

### Data Selection

The online forum platform Reddit provides a unique opportunity to study the effects of membership and activity patterns in various online communities on the risk of recurring use episodes [8,18,21]. Reddit is a public social news and discussion website where user-created content is organized into topic-based boards called *subreddits*. It accommodates >100,000 different subreddits where content is shared within a community of interest, such as about politics, business, parenting, medical conditions, sports, literature, music, video games, and life choices. This abundance of social groups allows us to simultaneously examine the effects of substance use, recovery, and non–drug-related online communities on the risk of a use episode during remission.

The subreddit r/OpiatesRecovery has >31,000 members and has provided individuals who wish to recover from OUD with recovery information and peer support since 2012. Some members make statements about their remission status on a regular basis by announcing the number of days they have not been using opioids. In combination with statements announcing a use episode, this provides us with information about the point in the recovery journey when a community member had a recurring use episode. Furthermore, anonymous but unique user IDs allow us to gather data on community members’ activities across the entire Reddit platform, providing behavioral information on engagement with online communities. Using behavioral information, problems with memory bias and differences in the definition of what is meant by a *group membership* can be circumvented. This is particularly important in light of the recent finding that online interactions are not spontaneously self-reported as social contact [42].

### Ethics Approval and Privacy Considerations

Before work commenced, the study received ethics approval from the University of Exeter's institutional review board (eCLESPsy001576), in line with the guidelines of the British Psychological Society and the American Psychological Association. This study used publicly accessible Reddit data for the analysis. Reddit usernames were used to collect quantitative data across different subreddits and were then replaced with anonymous participant numbers in the working data set. In this study, we do not report any user identifiable information to protect user privacy (eg, direct quotations and usernames); instead, we paraphrase quotations to illustrate our method.

### Data Collection and Preparation

**Figure 1:**
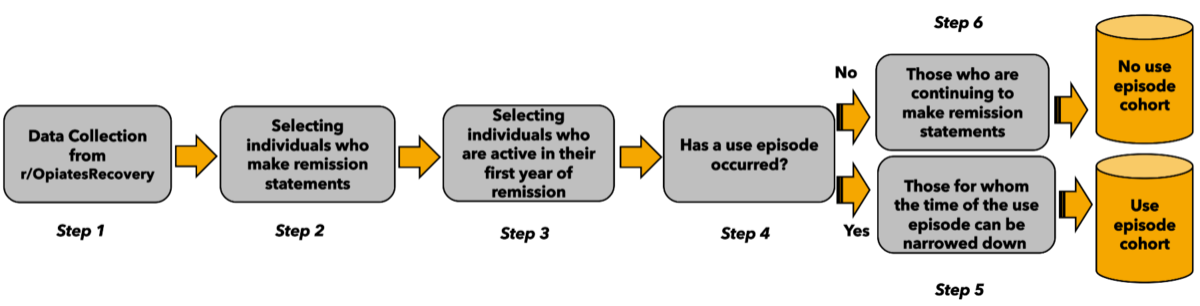
Diagram of data collection and preparation for identifying use episode and no use episode cohorts.

The steps involved in the data collection and preparation processes are illustrated in Figure 1. The study used publicly accessible data from r/OpiatesRecovery between February 2012 (when the group started) and June 2019 (the month of data collection).

After cleaning the data from adversarial content, bots, and so on, the initial data set contained 295,232 posts and comments from 18,125 individuals (step 1). Among these posts, we identified statements in which individuals announced their remission status (eg, “I’m 21 days clean”) and found 2950 instances of remission being announced by 1651 individuals (step 2). As we wished to study the activity of those in remission from OUD from the beginning of their current remission period, individuals whose self-reported time in remission exceeded their time on Reddit by >1 year were excluded from the analyses (eg, an individual who joined in 2015 but said in a post that they have been in remission since 2012). To allow sufficient information about each individual in our data set, we only retained data from those who posted at least 10 posts and comments in any subreddit for a minimum of 3 different months during the first year of remission. This resulted in 1081 individuals (step 3).

To identify which individuals had experienced a use episode, we examined statements commonly used to report such an episode (eg, “I started using again last night”). As we aimed to include as wide a sample as possible, we used inconsistent reporting of abstinence to identify those with a use episode. Where an inconsistent number of days without a use episode was reported (eg, “5 days without using” after announcing “21 days clean” a week earlier), a trained psychologist individually inspected remission status statements and found 51 additional individuals with a likely use episode. On the basis of statements and inconsistent reporting, we identified a *use episode* cohort of 335 individuals (step 4) among the 1081 individuals that regularly announced their remission progress. To ensure that survival analysis can estimate time correctly, we excluded those individuals for whom the time of the use episode could not be narrowed down to a specific month. For individuals whose remission statements only indirectly indicated a use episode, we used the month between the last report of a consistent period without a use episode and the announcement of a new period of remission (step 5). Individuals who had a consistent increase in remission days and did not make any statements about a use episode were grouped into the *no use episode* cohort. To ensure *noninformative* censoring, we only kept data from individuals in the *no use episode* cohort who were still active in r/OpiatesRecovery by the end of our data collection period in June 2019 (step 6). In total, our sample comprised 457 individuals from r/OpiatesRecovery, of whom 219 (47.9%) reported a use episode during remission, and 238 (52.1%) did not report a use episode.

### Labeling of Online Communities

To study the impact of distinct types of online groups on recovery progress, we categorized them into 3 types in line with the literature on the SIMOR: substance use, recovery, and non–drug-related groups. We considered subreddits that actively promoted substance use or discussed safe use as *substance use groups* (eg, r/Drugs, r/trees, and r/fentanyl). These also included legal substances such as alcohol consumption and tobacco smoking (eg, r/Cigarette, r/juul, and r/alcohol). Groups that discussed and supported recovery from addiction to any substance were considered recovery support communities (eg, r/narcoticsanonymous, r/quittingsmoking, and r/leaves). Both the description of the subreddit and posts within the community were considered when categorizing. Overall, 2 research assistants labeled the data, with the 2 authors providing guidance and reviewing the labels.

The participants in our data set were part of >4500 subreddits, which makes it impractical to label all of them. Therefore, we used a snowball approach where we started with r/Opiates and r/OpiatesRecovery, found similar subreddits to each of these, and then identified their category based on the content posted on the subreddit. We followed the same procedure for the newly identified subreddits, limiting our search to 4 rounds. We used Sayit [43], an online tool for finding subreddits similar to a specific one based on the number of mutual members. This procedure led to 1247 subreddits. Of these 1247 subreddits, 151 (12.11%) were labeled as substance use communities, 38 (3.05%) were labeled as recovery support communities, and 1058 (84.84%) were labeled as non–drug-related communities. Given that non–drug-related subreddits had a much higher prevalence, although we explicitly searched for subreddits on substance use and recovery support, we tested whether those who had not been labeled could be considered non–drug-related communities. To do so, we randomly selected 500 subreddits that had so far not been labeled and categorized them into substance use, recovery, and non–drug-related groups. Only 3.2% (16/500) were found to be substance use or recovery support groups. Therefore, it was decided that the risk of categorizing the remaining unlabeled forums as non–drug-related groups to the quality of the data was negligible.

To assess the effect of different types of groups on remission outcomes, researchers used 2 different operationalizations: the absolute number of groups of a particular type [20] and the proportion of a particular type of group relative to the total number of groups [11]. Assessing the absolute number of group memberships is in line with much of the social cure research that tends to find that a larger absolute number of group memberships is beneficial to health, mental health, and resilience [4]. In contrast, the SIMOR suggests that recovery from addiction is affected by the balance between substance use, recovery, and non–drug-related groups (ie, the proportion). Here, we use both operationalizations to examine the SIMOR and the wider social cure hypothesis.

For each participant, we counted the number of different subreddits in which they had posted at least once for each month from their self-reported remission start date until the first use episode or the last remission status announcement. On the basis of a labeled list of subreddits, we assessed the number of substance use, recovery, and non–drug-related group memberships for each month. In addition, we calculated the proportion of these group memberships by dividing membership into 1 of 3 categories (substance use, recovery, or non–drug-related) by the total number of group memberships in the same month (eg, number of substance use groups divided by number of all groups an individual has posted in). For each month, we also collected the number of posts and comments that a participant had contributed to the different online communities. Summary statistics for the sample are provided in Table 1, showing that participation in online communities far outstrips the number of groups that individuals who are in remission from a substance use disorder report in the offline world [11].

**Table 1:** Summary statistics of no use episode and use episode cohorts (N=457).

	All	No use episode	Use episode
Total sample, n (%)	457 (100)	238 (52.1)	219 (47.9)
Total posts, n (%)	237,435 (100)	137,030 (57.7)	100,405 (42.3)
Total subreddits, n (%)	4582 (100)	3513 (76.7)	2268 (49.5)
Posts per individual, median (range)	158 (1-15,805)	157 (1-15,805)	160 (3-7184)
Subreddits per individual, median (range)	14 (1-532)	18 (1-522)	11 (1-532)

### Data Analysis

The availability of longitudinal data up to a maximum of 6 years in an individual’s remission journey allowed us to predict the risk of a use episode as an outcome variable in a survival analysis (the step-by-step R code for the survival analysis is provided in Multimedia Appendix 1). The absolute number of substance use, recovery, and non–drug-related communities that an individual posted in at least once, as well as the proportion of these communities, served as predictor variables in our models. The number of posts and comments that an individual provided to these communities was statistically controlled for. This allowed us to test whether it is the number of group memberships, or an individual’s posting behavior, that is related to the risk of a use episode.

Survival analysis is a type of time-to-event analysis that has been widely adopted when the research interest is a combination of *whether* the event has occurred (binary outcome) and when it has occurred (continuous outcome). It provides unbiased survival estimates by using the information provided by individuals who have experienced the event (here, a use episode), as well as by those who have not (here, no use episode; so-called censored data). To explore the effects of various factors on the time to relapse, we used an extended Cox model [44]. This survival analysis regression method explores the relationship between the event of interest (the use episode in our study) and factors that affect the time at which the event occurs. This allowed us to study how survival probabilities change with changes in the studied factors. Unlike the basic Cox model, the extended version is designed to accommodate time-dependent variables; that is, variables whose values for a given participant may differ over time. Cox modeling does not make any assumptions about the statistical distribution of survival times, unlike most other statistical models, which makes it an appropriate choice for our research problem.

The data used in the study, the activity of individuals in terms of Reddit group membership over time, is provided as a supplementary file in Multimedia Appendix 1.

## Results

### Preliminary Results

Summary statistics (Table 2) of the number of substance use, recovery, and non–drug-related groups that individuals participated in at least once show that participation in non–drug-related groups is common among individuals in remission from OUD. They also indicate that those in the *use episode* cohort show significantly less engagement with non–drug-related groups than those in the *no use episode* cohort. Interestingly, there were no significant differences in the number of substance use and recovery groups, respectively, between the *no use episode* and the *use episode* cohorts.

**Table 2:** Summary statistics and significance tests for online community types by use episode and no use episode cohorts.

Communities	No use episode, median (range)	Use episode, median (range)	Significance test	
			*U* test	*P* value
Substance use	1 (0-10)	0 (0-28)	24,414.00	.20
Recovery	2 (1-11)	2 (1-11)	27,946.50	.16
Non–drug-related	16 (0-507)	8 (0-493)	22,036.00	.004

### Absolute Number of Group Memberships

In line with our hypotheses, we tested whether the absolute number of memberships in substance use, recovery, and non–drug-related groups affected the risk of a use episode during OUD remission. In line with SIMOR, we expect recovery and non–drug-related groups to decrease the risk of a use episode over time (hypotheses 1 and 2) and substance use groups to increase the risk of a use episode (hypothesis 3). The number of posts within these communities was controlled statistically. The statistics for the 3 separate survival analyses and the combined analysis are presented in Table 3.

**Table 3:** Effects of the absolute number of memberships in recovery, non–drug-related, and substance use online forums on the risk of a use episode during opioid use disorder remission.

Variable			*b* (SE)	*P* value	Odds ratio (95% CI)
**Separate models**					
	**Recovery models**				
		Number of posts (recovery)	0.002 (0.0008)	.04	1.0017 (5.61×10^−5^ to 0.003)
		Recovery memberships	−0.20 (0.08)	.009	0.8182 (−0.352 to −0.049)
	**Non–drug-related**				
		Number of posts (non–drug-related)	0.0004 (0.0002)	.005	1.0004 (0.0001 to 0.0006)
		Non–drug-related memberships	−0.05 (0.008)	<.001	0.9499 (−0.066 to −0.036)
	**Substance use models**				
		Number of posts (substance use)	0.002 (0.002)	.11	1.0020 (−0.0006 to 0.0046)
		Substance use memberships	−0.22 (0.07)	.002	0.8024 (−0.361 to −0.078)
**Combined model**					
	Number of posts (total)		0.0004 (0.0001)	.001	1.0004 (0.0001 to 0.0007)
	Recovery memberships		0.02 (0.07)	.73	1.02 (−0.108 to 0.154)
	Non–drug-related memberships		−0.06 (0.01)	<.001	0.94 (−0.073 to −0.040)
	Substance use memberships		0.08 (0.05)	.12	1.08 (−0.020 to 0.172)

In line with hypothesis 1, survival analysis showed that the number of online recovery groups an individual is a part of is negatively and significantly related to the risk of a use episode (*P*=.01), irrespective of the number of posts. Similarly, the higher the number of memberships in non–drug-related online groups, the lower the risk of a use episode (*P*<.001)*,* supporting hypothesis 2. However, in contrast to hypothesis 3, we found that the higher the absolute number of substance use group memberships, the lower (rather than higher) the risk of a use episode over time. The individual models also showed that the number of posts or comments made in each type of forum tends to increase the risk of a use episode. This effect was statistically significant for recovery and non–drug-related online forums.

When all 3 predictors were included in the same survival analysis, only the effect of the absolute number of non–drug-related online communities remained significant. Membership in non–drug-related groups has significant potential for reducing the risk of a use episode by 6% per additional non–drug-related group that an individual joins. Importantly, this effect is found when controlling for the number of posts or comments made, showing that the effect is not because of more active engagement in online communities. In fact, the total number of posts or comments was significantly and positively related to the occurrence of a use episode during remission, indicating a higher risk for those who contributed more frequently.

### Proportion of Group Memberships

Next, we tested the same 3 hypotheses but with the number of group memberships in a particular type of group (substance use, recovery, or non–drug-related) relative to the number of total Reddit communities in which an individual participated (ie, the proportion). Again, we statistically controlled for the total number of posts or comments that contributed to the respective type of group. The statistics for the 3 separate survival models are presented in Table 4.

Survival analysis showed an unexpectedly positive and significant effect of the proportion of recovery group memberships on the risk of relapse. Being part of a higher proportion of online recovery subreddits significantly increases the likelihood of an individual reporting a use episode over time (*P*<.001). This finding does not support hypothesis 1. The model also showed a marginally significant positive effect of the proportion of substance use groups on the risk of a use episode (*P*=.06). In line with hypothesis 3, this finding suggests that a higher proportion of substance use groups may increase the risk of a use episode during remission. In contrast, being part of a higher proportion of non–drug-related groups significantly decreases the risk of a use episode (*P*<.001), in line with hypothesis 2.

**Table 4:** Effects of the proportion of memberships in recovery, non–drug-related, and substance use online forums on the risk of a use episode during remission.

Variable		*b* (SE)	*P* value	Odds ratio (95% CI)
**Recovery models**				
	Number of posts (recovery)	0.001 (0.001)	.29	1.00 (−0.0008 to 0.0027)
	Recovery memberships (%)	1.61 (0.20)	<.001	5.00 (1.219 to 2.002)
**Non–drug-related models**				
	Number of posts (non–drug-related)	−0.001 (0.001)	.30	1.00 (−0.003 to 0.0009)
	Non–drug-related memberships (%)	−1.39 (0.26)	<.001	0.25 (−1.900 to −0.879)
**Substance use models**				
	Number of posts (substance use)	−0.004 (0.002)	.38	1.00 (−0.011 to 0.004)
	Substance use memberships (%)	1.16 (0.60)	.06	3.20 (−0.062 to 2.387)

## Discussion

### Principal Findings

Using naturally occurring data from the popular online platform Reddit, we examined the risk of a use episode over several years of remission from OUD. Our study tested predictions by the SIMOR that recovery and non–drug-related group memberships sustain remission, whereas substance use groups undermine it. Here, we specifically tested the SIMOR in an *online* environment to provide empirical evidence for the effects of online community memberships that go beyond membership in a single online recovery support group.

Our findings show that a higher number of memberships in non–drug-related online groups are associated with a lower risk of use episodes during OUD remission. The more online non–drug-related groups an individual recovering from OUD becomes part of, the lower the risk of a use episode over time. This effect persisted when membership in recovery and substance use groups was accounted for. These findings support the SIMOR’s focus on building non–drug-related group memberships to sustain remission [1]. They also support predictions by the wider social cure literature, which suggests that social groups deliver health benefits and create resilience [4].

In contrast to much of the literature, we found mixed support for membership in recovery support groups. The results indicate that a higher absolute number of memberships in recovery support groups is associated with a reduction in the risk of a use episode but only when other types of group membership are not controlled for. Furthermore, we found a significant increase in the risk for those with a higher proportion of recovery groups among all their Reddit groups.

We offer 2 explanations for this result. First, engaging with several recovery support forums unrelated to opioid remission may indicate that the individual is dealing with polysubstance dependence or has been using multiple substances to self-medicate health issues (eg, chronic pain) or underlying mental health problems (eg, depression and anxiety). Recovering from multiple substances in addition to opioids may increase the chances of a use episode because of the higher risk to mental and physical health [45]. However, this explanation cannot account for the finding that the absolute number of recovery support group memberships reduced the risk of a use episode (or was unrelated to risk when other group memberships were accounted for). A second possible explanation is that a narrow focus on online recovery support groups may be detrimental to sustained remission when online activities do not include participation in non–drug-related groups. Investing primarily in recovery support groups (ie, having a high proportion of recovery support group memberships relative to other memberships) appears to undermine building a resilient social self that incorporates a variety of social identities derived from valued group membership in different spheres of life. This points toward a need for future research to examine the extent to which membership in (online) recovery support groups may reduce engagement with non–drug-related communities and the effects of this on the risk of recurring use episodes and other remission outcomes. Examining the effects of recovery support group membership in isolation may risk missing wider, potentially detrimental effects.

Furthermore, our analysis uses data across several years, thereby examining a longer period of recovery than is usually investigated in the literature, which tends to focus on 6 months to 1 year after treatment. Therefore, it is possible that our findings reflect the importance of non–drug-related identities that replace a *recovery* identity in the long term, as suggested by the SIMOR [1]. Hence, our work provides the first indication that the recovery journey needs to continue beyond building recovery support group memberships and that online communities can play a part in providing a diverse range of non–drug-related group memberships.

We also found inconsistent support for the proposed detrimental effect of substance use groups on the risk of a use episode during remission. Memberships in online communities that promote substance use did not show the expected effect of increasing the risk of a use episode. Instead, we found a significant negative effect of the absolute number of substance use communities on the risk of a use episode. This effect disappeared when other group memberships were controlled for. However, we also found a marginally significant effect that a higher proportion of substance use community memberships increases the likelihood of a use episode. Together, this inconsistent pattern does not unequivocally support the assumption by the SIMOR that substance use group memberships undermine long-term remission.

This finding is surprising as substance use groups on Reddit share pictures of drugs and paraphernalia. Such pictures have been linked to cravings in individuals with substance use disorder [46]. However, our study did not exclusively focus on substance use groups linked to opioids. Those who participate in substance use communities may seek alternative treatments to avoid opioid use [47]. Furthermore, some researchers have challenged the assumption by the SIMOR, as well as similar models, that substance use groups are necessarily detrimental. For instance, qualitative research has shown that groups (and individuals) associated with continuing substance use can provide significant recovery support through information [48] and trusted and emotionally significant friendships [40]. Future research is needed to provide a clearer, as well as a more differentiated, picture of substance use group memberships and their part in the recovery journey.

Importantly, we controlled for posting activity throughout to test whether the number of group memberships or the activity level drives effects. Interestingly, posting activity was positively related to the risk of a use episode, suggesting that those who posted, or commented, more frequently were more likely to report a use episode. Surprisingly, this effect was significant for both recovery and non–drug-related online communities, indicating that this effect was not because of an increased discussion of drug-related issues. Future research should examine whether posting frequency is an early warning sign of recurring use episodes or whether other variables related to posting activities act as risk factors.

### Limitations and Future Research

To test the SIMOR and extend previous research on the effects of online community memberships on the risk of a use episode during OUD remission [21], we categorized subreddits into substance use, recovery, and non–drug-related groups. Our discussion of the effects of recovery groups and substance use groups indicates that such a classification might be too simplistic to capture the effects of specific groups on the risk of recurring use episodes. The supposedly neat categorization into these 3 types of groups by the SIMOR has already been criticized elsewhere [40,41,49]. Although such labeling of groups provides some benefits in terms of public health messaging, it may not adequately reflect the complexities on the ground.

Another limitation is the lack of demographic information and other relevant information in our sample. As a result, we cannot statistically control for potential confounding variables such as previous opioid use severity, treatment uptake, comorbidity, and other relevant variables. For instance, individuals who manage to join more online groups during their remission from OUD may have personal skills (eg, self-confidence) and privileges (eg, free time and unrestricted internet access) that allow them to engage with more online communities.

The sample size of our study was smaller than those of other studies that used computational approaches. However, the sample size is still larger than that of the vast majority of studies in the area of social group effects on remission outcomes. Furthermore, sample size is not an indicator of the validity of the findings. It is increasingly being recognized that an important challenge when using online data for clinical or social research questions is that validity is established across all components: a high-quality sample, valid measures, and appropriate statistical methods. As outlined in the *Methods* section, the sample size was determined by methodologically justified steps to ensure valid conclusions. For instance, rather than including all individuals who posted in r/OpiatesRecovery and did not report a use episode into the *no use episode* cohort, we ensured that we had a clear indication from remission status statements that no use episode had occurred where inconsistent remission status statements were made, and a trained psychologist reviewed the case. Similarly, we invested time in labeling a large number of subreddits not only based on their name or mission statement but also by taking the posts in the subreddits into account.

Importantly, our research found that the activity level in communities was unrelated to the risk of a use episode or increased risk rather than lowering it. However, we do not have data for individuals who are entirely passive members of a community; that is, those who read posts but never or rarely post themselves (sometimes referred to as *lurkers*). The CEO of Reddit, Steve Huffman, estimates that two-thirds of the members may fall into this category [50]. Recent research shows that active participation in face-to-face groups is not an attractive or viable option for everyone [14]. Therefore, understanding whether *lurking* is enough to provide a person with a sense of social identity and group resources (eg, advice and shared experience) is a relevant (although technically challenging) avenue for future research.

### Practical Implications

Online communities provide a new methodological way of studying remission from substance use disorders. The public and anonymous nature of online communities allows us to examine longitudinal data of those who are in remission over several years, including data from individuals that may be difficult to recruit for traditional surveys or interview studies (eg, those who do not access therapy through the health care system). Much of the literature on social group effects on remission relies on cross-sectional data, with only a few studies testing longitudinally, rarely for more than 6 months or a year at best. Sample sizes tend to be small (often N=20-50 for smaller, cross-sectional studies, with N=200-350 for large studies) and often stem from therapeutic communities or peer-support groups (eg, AA or Narcotics Anonymous), thereby limiting findings to those who already successfully access formal support. Examining online community behavior, such as participation in different online forums, also allows us to observe real-world behavior rather than having to rely on the memory and subjective definitions of participants in survey studies and interviews.

Online communities have the potential to benefit those who are in remission from substance use disorder by providing relatively easy access to social groups. Our findings suggest that fairly minimal engagement with non–drug-related groups can increase resilience during recovery over a long period. Although online communities are not universally accessible because of the requirement for an internet connection and basic literacy and computer skills, they create fewer barriers than many offline groups [14]. They may also provide a sense of continuity to those who need to move their location.

Importantly, our research indicated that an exclusive or narrow focus on recovery support communities may increase the risk of recurring use episodes. Platforms and mutual help communities that offer online support to those in substance use remission but are not part of a wider platform (eg, Reddit) may wish to flag non–drug-related online communities to their members to enable them to build wider social group memberships and avoid a narrow focus on recovery support groups, particularly in the longer term.

### Conclusions

Opioid addiction is one of the most pressing public health issues of the day and was declared a national health emergency by the US government in 2017 [51], with an average of 128 overdose deaths from opioids every day in the United States alone [52]. Worldwide, 118,000 deaths in 2015 were directly associated with OUD [53]. Access to therapy and recovery support groups can prove difficult for several reasons, such as a lack of funding, stigma, and personal circumstances. Online groups, such as forums on popular platforms like Reddit, may provide a lifeline for those who are in remission from OUD. Providing evidence-based support for the use of online groups during OUD remission is an important public health task. Here, we provide the first evidence that online forums that are unrelated to substance use and recovery advice can provide social capital that significantly reduces the risk of a use episode across several years significantly—by as much as 6% per additional non–drug-related online community. Our results also suggest that more research is needed to understand the circumstances under which ties with substance use groups may not pose a risk to sustained remission from OUD. Similarly, further research is needed to understand the circumstances under which a narrow focus on online recovery support groups may be harmful during OUD remissions.

## Appendix

Multimedia Appendix 1 Data collection and survival analysis.

## Abbreviations

AA: Alcoholics Anonymous
OUD: opioid use disorder
SIMOR: Social Identity Model of Recovery
