# Supplementary material for: Effects of Substance Use, Recovery, and Non–Drug-Related Online Community Participation on the Risk of a Use Episode During Remission From Opioid Use Disorder: Longitudinal Observational Study
Source: J Med Internet Res. 2022 Aug 22;24(8):e36555. doi: 10.2196/36555 (PMC9446138; doi:10.2196/36555)
Supplement: Multimedia Appendix 1 [file jmir_v24i8e36555_app1.docx]

Effects of Substance Use, Recovery, and Non–Drug-Related online Community Participation on the Risk of a Use Episode During Remission From Opioid Use Disorder (OUD): Longitudinal Observational Study : Appendix 1

Data Collection and Survival Analysis

We collected data from r/OpiatesRecovery through Google BigQuery (<https://cloud.google.com/bigquery>). We then used Pushshift Reddit Api (<https://github.com/pushshift/api> to collect the public Reddit participation history for each participant in our dataset, from their recovery start date until the last consistent recovery announcement. The final Data for this study which includes the users’ activity over Reddit can downloaded from our OSF repository <https://osf.io/u74hc/>, along with the step-by-step R code for the survival analysis <https://osf.io/dtg2u/> .
